# Walking Speed and Hand Grip Strength as Predictors of Hypertension Risk in Older Adults: The Impact of Insulin Resistance

**DOI:** 10.1111/ggi.70225

**Published:** 2025-10-21

**Authors:** On Lee, DooYong Park

**Affiliations:** ^1^ Department of Sport Science Korea Institute of Sports Science Seoul Korea; ^2^ Department of Physical Education Seoul National University Seoul Korea

**Keywords:** aged, cohort studies, hand strength, hypertension, insulin resistance, walking speed

## Abstract

**Aim:**

This study aimed to investigate the independent associations of walking speed (WS) and hand grip strength (HGS) with the risk of hypertension in older adults.

**Methods:**

We analyzed data from 1890 older adults participating in a Korean community‐based cohort study. Hypertension was defined as systolic blood pressure ≥ 140 mmHg, diastolic blood pressure ≥ 90 mmHg, self‐reported physician diagnosis, or current antihypertensive medication use. WS was measured using a 4‐m walking test (m/s), and HGS was assessed with a hydraulic hand dynamometer (kg/BMI). Multivariable logistic regression models were used to estimate odds ratios (OR) and 95% confidence intervals (CI), adjusting for potential confounders including age and gender.

**Results:**

After controlling for confounding variables, participants in the high WS group had a 46% lower odds of hypertension compared with those in the low WS group (OR = 0.52, 95% CI = 0.29–0.94). Notably, among participants with low insulin resistance, higher WS was associated with a markedly reduced risk of hypertension (OR = 0.35, 95% CI = 0.14–0.85). Additionally, compared with the WS low/HGS low group, those in the WS high/HGS low and WS high/HGS high groups demonstrated a 55% (OR = 0.45, 95% CI = 0.22–0.92) and 54% (OR = 0.46, 95% CI = 0.23–0.95) lower odds of hypertension, respectively, with differences observed according to insulin resistance status.

**Conclusion:**

Our findings indicate that WS is an effective predictor of hypertension risk in older adults, particularly among individuals with lower insulin resistance.

## 1 Introduction

Hypertension, defined as a medical condition that increases the risk of cardiovascular and cerebrovascular events, affects one‐quarter of the global population [1]. Despite advances in management and treatment, the mortality risk and economic burden associated with hypertension remain high [2]. In fact, according to data released by the American College of Cardiology and the American Heart Association, 48.1% of American adults have hypertension [3]. Additionally, the 2023 National Health and Nutrition Examination Survey in Korea reported that the prevalence of hypertension is 33.6% among adults aged ≥ 19 years and rises to 63.5% among older adults aged ≥ 65 years, indicating an increased vulnerability in the elderly [4]. Such high prevalence worldwide underscores the importance of primary prevention of hypertension as a national public health priority, and previous studies have highlighted the critical role of lifestyle modifications in hypertension management [5].

Measures of physical function, such as walking speed (WS) and hand grip strength (HGS), offer rapid, objective alternatives to traditional vital sign monitoring and have been emphasized as important indicators in disease prediction [6]. These measures are also closely associated with hypertension [7, 8]. Notably, while HGS assesses the force of muscle contraction through a simple test, WS integrates several components—including lower body strength, balance, and coordination—which may result in a stronger association with health outcomes when combined [9].

To date, most studies evaluating the relationship between HGS and WS with health outcomes have predominantly focused on mortality risk [10, 11]. Only a few studies have examined the association with hypertension risk by investigating either HGS [8, 12] or WS [13, 14] independently. One study even evaluated the interaction between HGS and WS on hypertension risk; however, this study relied solely on self‐reported WS categorized into three levels (slow, average, and brisk), highlighting limitations in objective measurement. Moreover, while that study suggested that insulin resistance is an important mediator between hypertension, muscle strength, and WS, it did not incorporate insulin resistance in the analysis of various metabolic issues [15].

Therefore, this study aims to investigate the independent associations between WS, HGS, and hypertension risk, as well as the interactive effect of WS and HGS on hypertension risk. In addition, we compare these associations across different levels of insulin resistance.

## 2 Methods

### 2.1 Study Participants

This study utilized data from the Korean Genome and Epidemiology Study (KoGES), a large‐scale cohort survey designed to investigate health and lifestyle factors for the prevention of chronic disease among Koreans. We selected general adults residing in Ansan City, Gyeonggi Province, who participated in the baseline survey conducted in 2011–2012. Participants were aged 55–82 years. A total of 3052 individuals were initially recruited via telephone, mail, and home visits. After excluding participants with cardiovascular disease (*n* = 26) and those with missing data on HGS, WS, or any variables known to affect hypertension (including age, gender, regular exercise participation, skeletal muscle mass, sleep duration, estimated glomerular filtration rate [eGFR], high‐sensitivity C‐reactive protein [hs‐CRP], triglycerides, total cholesterol, alcohol intake, smoking status, income level, body mass index [BMI], and homeostasis model assessment of insulin resistance [HOMA‐IR]) (*n* = 1136), a final sample of 1890 participants was included in the analysis. This study was approved by the Institutional Review Board of Korea University Ansan Hospital and the Seoul National University Bioethics Committee (IRB No. E2501/004–006), and written informed consent was obtained from all participants after explaining the study's purpose and procedures.

### 2.2 Measurement Variables

#### 2.2.1 Hypertension Prevalence

Hypertension was defined in accordance with previous studies as systolic blood pressure ≥ 140 mmHg, diastolic blood pressure ≥ 90 mmHg, self‐reported diagnosis of hypertension, or current use of antihypertensive medication [16, 17].

#### 2.2.2 HGS and WS

HGS was measured using a hydraulic hand dynamometer (SAEHAN, Korea) while participants were seated with their arms maintained at a 90° angle. Measurements were taken alternately from both hands three times, and the average of the maximum values for each hand was used. Recent studies have demonstrated that relative HGS is a more effective measurement than absolute HGS for assessing muscle weakness and metabolic syndrome. Therefore, we decided to use absolute HGS standardized by BMI in this study [18]. Based on prior literature, participants were classified into two quantiles by gender: those in the lower 50% were classified as “Low,” and those in the upper 51%–100% as “High” [19]. WS was measured as the time taken to walk a 4‐m distance, with the fastest of two trials recorded for analysis. Based on previous studies, WS was categorized as “Low” if it was below 0.8 m/s and “High” if it was 0.8 m/s or greater [20].

#### 2.2.3 Blood Biomarkers

All participants were instructed to fast for at least 8 h prior to blood collection. On the day of the examination, blood samples were collected and processed onsite using a centrifuge, then sent to the Seoul Clinical Laboratory where they were analyzed using an ADVIA 1800 auto analyzer (Siemens, USA). From these assays, we utilized results for hs‐CRP, creatinine, total cholesterol, triglycerides, glucose, and insulin. Serum samples were stored collectively by the Genomic Research Team at the Korea Disease Control and Prevention Agency.

eGFR was calculated using serum creatinine according to the Modification of Diet in Renal Disease (MDRD) study formula and was analyzed as a continuous variable [21]: in mL/min per 1.73 m^2^ = 175 × serum creatinine[exp (−1.154)] × age [exp(−0.203)] × [0.742 if female] × [1.21 if black]. HOMA‐IR was calculated using the formula as described in previous research [22]: fasting insulin × fasting blood glucose/405.

#### 2.2.4 Questionnaire and Other Variables

Face‐to‐face interviews were conducted by trained surveyors, and questionnaires were reviewed and revised on the day of the survey to ensure data completeness. Height (cm) and weight (kg) were measured once, and skeletal muscle mass was assessed using InBody 3.0 (Biospace, Korea). BMI was calculated as weight (kg) divided by height squared (m^2^) and used to classify obesity levels. Blood pressure was measured using a standardized sphygmomanometer, and the average of the measurements taken from the right and left arms was used for analysis. Alcohol consumption and smoking status were determined using a standardized questionnaire developed for KoGES. For alcohol, responses were categorized as “never drinker,” “past drinker,” or “current drinker.” For smoking, responses were categorized as “never smoker,” “past smoker,” or “current smoker.” Sleep duration was calculated as the weighted average of reported weekday and weekend sleep hours using the formula: ([weekday hours × 5] + [weekend hours × 2])/7. Regular exercise participation was determined based on responses to the question “Do you engage in exercise that causes you to sweat on a regular basis?” with responses classified as “No” (non‐participation) or “Yes” (participation). Monthly household income was recorded and categorized as “< 1 million KRW,” “1–1.99 million KRW,” “2–2.99 million KRW,” “3–3.99 million KRW,” and “≥ 4 million KRW.” Occupational classification was determined via responses to “What is your current occupation?” using a 14‐category system and subsequently grouped as “professional/managerial/clerical,” “sales/service,” “agricultural/forestry/fisheries/technical,” “household/work,” and “others.”

### 2.3 Statistical Analysis

Data analysis was performed using STATA/IC 14.1 (STATA Corp., College Station, TX, USA). Descriptive analyses for continuous variables were conducted by computing means ± standard deviations, and categorical variables were analyzed using *χ* ^2^ tests and reported as percentages (see Table 1). To compare differences in hypertension prevalence, WS, and HGS levels across groups, one‐way ANOVA with Bonferroni post hoc tests was applied (*p* < 0.05).

**TABLE 1:** Baseline characteristics of study participants by hypertension status.

Characteristics of risk factor	Non‐hypertension (*n* = 705)	Hypertension (*n* = 1185)	*p*
Age (years)	69.26 ± 6.22	70.59 ± 6.12	< 0.001
Sex (male %)	47.23	38.99	< 0.001
BMI (kg/m^2^)	23.15 ± 3.13	24.84 ± 3.31	< 0.001
Lean body mass (kg)	38.90 ± 7.29	39.02 ± 7.77	0.758
Sleep duration (hour/day)	6.55 ± 1.35	6.64 ± 1.35	0.149
eGFR (mL/min per 1.73m^2^)	94.67 ± 20.33	88.21 ± 22.02	< 0.001
hs‐CRP (mg/dL)	1.68 ± 4.55	1.69 ± 4.13	0.950
Triglyceride (mg/dL)	122.17 ± 73.04	135.41 ± 78.11	< 0.001
Total cholesterol (mg/dL)	182.94 ± 32.59	177.27 ± 33.44	< 0.001
HOMA‐IR	1.90 ± 1.10	2.47 ± 1.88	< 0.001
Current alcohol consumption (%)	36.74	31.48	0.037
Current smoking (%)	13.19	9.45	0.017
Regular exercise participation (%)	27.23	24.98	0.279
Low income status (< 1 million won %)	59.82	66.90	0.052
HGS (kg/BMI)	1.11 ± 0.42	0.98 ± 0.37	< 0.001
WS (m/s)	1.31 ± 0.25	1.25 ± 0.29	< 0.001

Abbreviations: BMI, body mass index; eGFR, estimated glomerular filtration rate; HGS, hand grip strength; HOMA‐IR, homeostatic model assessment for insulin resistance; hs‐CRP, human serum‐C‐reactive protein; WS, walking speed.

Logistic regression analysis was used to calculate odds ratios (OR) and 95% confidence intervals (CI) for the associations between WS (categorized as low versus high) and HGS (categorized as low versus high) with hypertension risk (Table 2), including separate analyses stratified by insulin resistance levels (Table 3).

**TABLE 2:** Odds ratio of hypertension risk according to HGS and WS.

Characteristics of risk factors	Total *n* (*n* = 1890)	Hypertension *n* (*n* = 1185)	Model 1 OR (95% CI)	Model 2 OR (95% CI)
WS				
Low (< 0.8 m/s)	80	63	1.00 (Reference)	1.00 (Reference)
High (≥ 0.8 m/s)	1810	1122	**0.52** * **(0.29, 0.94)**	**0.54** * **(0.30, 0.97)**
*p*			0.033	0.041
HGS				
Low	944	650	1.00 (Reference)	1.00 (Reference)
Male (0.38 ~ 1.38 kg/BMI)				
Female (0.22 ~ 0.76 kg/BMI)				
High	946	535	0.99 (0.79, 1.25)	1.00 (0.79, 1.27)
Male (1.38 ~ 2.40 kg/BMI)				
Female (0.76 ~ 1.52 kg/BMI)				
*p*			0.993	0.938

*Note:* Multivariable model 1 was adjusted for age, sex, sleep duration, lean body mass, triglyceride, total cholesterol, eGFR, hs‐CRP, alcohol intake, smoking status, income status, and exercise participation. Model 2 was adjusted for Model 1 variables plus HOMA‐IR. Incidence density = case/person‐year×1000.

Abbreviations: BMI, body mass index; eGFR, estimated glomerular filtration rate; HGS, hand grip strength; HOMA‐IR, homeostatic model assessment for insulin resistance; hs‐CRP, human serum‐C‐reactive protein; OR, odds ratio; WS, walking speed.

* *p* < 0.05.

**TABLE 3:** Odds ratio of hypertension risk according to HGS and WS by IR.

Characteristics of risk factors	Low IR OR (*n* = 812)	High IR OR (*n* = 1078)
WS		
Low (< 0.8 m/s)	1.00 (Reference)	1.00 (Reference)
High (≥ 0.8 m/s)	**0.35** * (**0.14, 0.85**)	0.79 (0.36, 1.74)
*p*	0.021	0.572
HGS		
Low	1.00 (Reference)	1.00 (Reference)
Male (0.38 ~ 1.38 kg/BMI)		
High	0.98 (0.69, 1.39)	1.02 (0.74, 1.41)
Male (1.38 ~ 2.40 kg/BMI)		
Female (0.76 ~ 1.52 kg/BMI)		
*p*	0.934	0.887

*Note:* Multivariable model was adjusted for age, sex, sleep duration, lean body mass, triglyceride, total cholesterol, eGFR, hs‐CRP, alcohol intake, smoking status, income status, exercise participation, HOMA‐IR.

Abbreviations: BMI, body mass index; eGFR, estimated glomerular filtration rate; HGS, hand grip strength; HOMA‐IR, homeostatic model assessment for insulin resistance; hs‐CRP, human serum‐C‐reactive protein; IR, insulin resistance; OR, odds ratio; WS, walking speed.

* *p* < 0.05.

**FIGURE 1:**
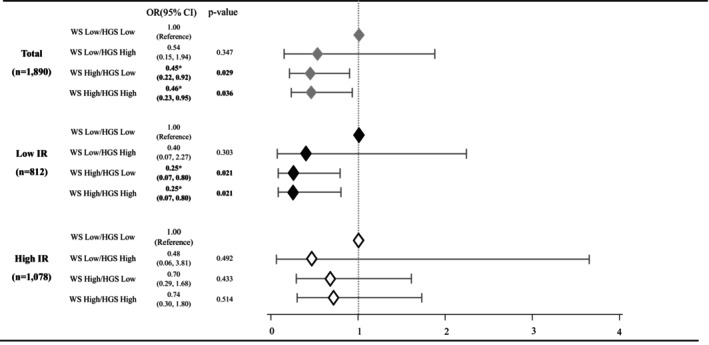
Odds ratio of hypertension according to joint association between WS and HGS by IR level. Multivariable Model was adjusted for age, sex, sleep duration, lean body mass, triglyceride, total cholesterol, eGFR, hs‐CRP, alcohol intake, smoking status, income status, HOMA‐IR and exercise participation. BMI, body mass index; eGFR, estimated glomerular filtration rate; HGS, hand grip strength; HOMA‐IR, homeostatic model assessment for insulin resistance; hs‐CRP, human serum‐C‐reactive protein; IR, insulin resistance; OR, odds ratio; WS, walking speed. *p < 0.05.

Furthermore, the interaction between WS and HGS with respect to hypertension risk was evaluated using logistic regression analysis, stratified by insulin resistance levels (Figure 1). All logistic regression models were adjusted for potential confounders known to affect both physical activity and hypertension, including age, gender, sleep duration, eGFR, hs‐CRP, alcohol intake, smoking status, income level, BMI, HOMA‐IR, regular exercise participation, triglycerides, and total cholesterol. All statistical tests were two‐tailed, with significance set at *p* < 0.05.

## 3 Result

Our final analytic sample consisted of 1890 adults (mean age 70.09 ± 6.19 years) from the baseline survey, including 705 individuals without hypertension and 1185 with hypertension. Demographic characteristics are presented in Table 1. Participants with hypertension were older; they had a higher proportion of females, elevated BMI, higher triglyceride levels, and increased HOMA‐IR, while exhibiting a lower proportion of males, lower eGFR, reduced total cholesterol, and lower rates of smoking, alcohol consumption, HGS, and WS compared to those without hypertension. No significant differences were observed between the groups in muscle mass, sleep duration, hs‐CRP, regular exercise participation, or income level.

The associations between WS, HGS, and hypertension risk are detailed in Table 2. In Model 1, which adjusted for multiple confounders including age and gender, participants in the high WS group had 48% lower odds of hypertension compared with those in the low WS group (OR = 0.52, 95% CI = 0.29–0.94). Similar results were obtained in Model 2, which further adjusted for HOMA‐IR (OR = 0.54, 95% CI = 0.30–0.97). In contrast, no statistically significant association was observed between HGS and hypertension risk across all models (Model 1: *p* = 0.993; Model 2: *p* = 0.938).

Table 3 presents the associations between WS, HGS, and hypertension risk stratified by insulin resistance. Among participants with low insulin resistance, higher WS was significantly associated with reduced hypertension risk (OR = 0.35, 95% CI = 0.14–0.85), whereas HGS showed no significant association (*p* = 0.934). In participants with high insulin resistance, neither WS nor HGS was significantly related to hypertension risk (WS: *p* = 0.572; HGS: *p* = 0.887).

Figure 1 illustrates the interaction between WS and HGS on hypertension risk. Compared with the group with both low WS and low HGS, the WS high/HGS low and WS high/HGS high groups experienced 55% (OR = 0.45, 95% CI = 0.22–0.92) and 54% (OR = 0.46, 95% CI = 0.23–0.95) lower odds of hypertension, respectively, with these reductions being even more pronounced among participants with low insulin resistance (WS High/HGS Low: OR = 0.25, 95% CI = 0.07–0.80; WS High/HGS High: OR = 0.25, 95% CI = 0.07–0.80).

## 4 Discussion

In this study, after adjusting for various confounding variables and insulin resistance, a faster WS was independently associated with a reduced odds ratio for hypertension compared to a slower WS, especially among participants with low insulin resistance. Moreover, regardless of HGS levels, a faster WS was consistently linked to a decreased risk of hypertension.

Numerous studies have underscored the utility of WS and HGS as objective markers for predicting disease risk. For instance, a prospective UK Biobank study of 406,834 participants demonstrated that the combined assessment of WS and HGS provided superior cardiovascular disease risk prediction compared to evaluating each measure individually [6]. Additional research has indicated that integrating these measures enhances the prediction of adverse health outcomes and highlights the close interrelationships among muscle strength, WS, and metabolic disturbances [9]. Indeed, previous work examining the association between HGS, WS, and hypertension found that groups with both high HGS and fast WS exhibited the lowest hypertension risk (HR = 0.36; 95% CI: 0.25–0.52) compared to those with low HGS and slow WS [15].

Conversely, several prior studies have reported that HGS alone is not consistently associated with hypertension risk [12, 13]. In contrast, faster WS has repeatedly emerged as a significant predictor of reduced hypertension risk [12, 13]. For example, individuals with faster WS were found to have a 1.37‐fold lower risk of developing hypertension (HR = 0.64; 95% CI: 0.41–0.99) compared to those with slower WS [23], and other studies have similarly shown that WS is a more effective predictor of hypertension than HGS (HR = 0.06; 95% CI: 0.11–0.30) [13].

In Table 2, the consistent inverse association observed between WS and hypertension risk OR, independent of other confounders and insulin resistance, suggests that a faster WS serves as a marker of superior cardiopulmonary fitness and overall health [24]. Enhanced cardiopulmonary function improves arterial elasticity and insulin sensitivity, while nitric oxide produced by endothelial cells promotes vasodilation, thereby reducing blood pressure [25]. These findings are consistent with our results in Table 1, which showed that participants without hypertension had a WS that was, on average, 0.06 m/s faster and a HOMA‐IR value that was 0.57 units lower than those with hypertension. Thus, given the close association between hypertension and microvascular disease [26], an increase in WS—reflecting improved vascular dilation and enhanced insulin sensitivity—may serve as a robust predictor of hypertension risk [13].

However, in Table 3 and Figure 1, no significant association between WS and hypertension risk OR was observed among participants with high insulin resistance. This may be attributed to the diverse adverse effects on vascular health that arise when insulin resistance exceeds a critical threshold. Previous research indicates that in groups with elevated insulin resistance, impaired vasodilation and increased renal sodium reabsorption contribute to a heightened risk of hypertension [27]. Moreover, high circulating insulin levels linked to insulin resistance can stimulate sympathetic nervous system activity and elevate inflammatory cytokine production, thereby increasing peripheral vascular resistance and further promoting hypertension; these adverse metabolic effects underscore the role of insulin resistance as a key marker of metabolic dysfunction [28]. Consequently, the detrimental vascular effects associated with high insulin resistance [27, 28] may overshadow the protective effect of a faster WS [23], resulting in a lack of significant association in this subgroup.

In Figure 1, irrespective of HGS levels, a faster WS is consistently associated with reduced odds of hypertension. This finding suggests that WS is a more effective predictor of disease risk than HGS. Recent review studies have indicated that WS measurement alone is a valuable tool for screening for frailty [29], and comparative analyses have shown that WS provides superior prognostic information for mortality compared to HGS, which is primarily used to define sarcopenia and may have limitations in predicting disease risk [10]. Therefore, WS appears to be a more effective indicator than HGS for predicting hypertension, a condition that is closely linked to multiple physiological abnormalities associated with frailty [13].

This cross‐sectional study, which examined the association between the interaction of HGS and WS with hypertension risk across different levels of insulin resistance, has important implications; however, it is not without limitations. First, we were unable to adjust for dietary variables, such as salt intake and processed food consumption, which may influence hypertension. Nevertheless, we enhanced the reliability of our findings by adjusting for lifestyle factors and various blood markers known to affect hypertension risk. Second, given the cross‐sectional nature of this study, it is difficult to establish a causal relationship between HGS, WS, and hypertension risk; future research should aim to validate these findings with longitudinal follow‐up of groups defined by the interaction between HGS and WS. Third, our study sample was drawn from a specific region in Korea, which may limit the generalizability of the results to the broader Korean adult population. Consequently, additional studies incorporating nationally representative samples are needed to expand upon our findings.

## 5 Conclusion

This study confirmed that WS is an effective predictor of hypertension risk, with the association being even more pronounced among individuals with lower levels of insulin resistance. Based on these findings, measuring WS in individuals with low insulin resistance could serve as a straightforward tool for predicting metabolic disorders such as hypertension. Furthermore, our results underscore the need for national and corporate initiatives to promote walking and sustained aerobic exercise programs to help reduce the risk of hypertension.

## Author Contributions

Study concept and design: D.P. and O.L.; acquisition of data: D.P.; analysis and interpretation of data: D.P. and O.L.; drafting of the manuscript: D.P. and O.L.; critical revision of the manuscript: D.P. and O.L.; statistical analysis: D.P.; administrative, technical, or material support: O.L.; and study supervision: O.L.

## Ethics Statement

This study was approved by the Institutional Review Boards of Korea University Ansan Hospital and Seoul National University (IRB No. E2501/004‐006).

## Consent

The authors have nothing to report.

## Conflicts of Interest

The authors declare no conflicts of interest.

## Data Availability

The data that support the findings of this study are available on request from the corresponding author. The data are not publicly available due to privacy or ethical restrictions.
